# Moral Elevation Online Intervention for Veterans Experiencing Distress Related to Posttraumatic Stress Disorder and Moral Injury (MOVED): Pilot Trial of a 4-Week Positive Psychology Web-Based Intervention

**DOI:** 10.2196/39894

**Published:** 2023-03-24

**Authors:** Adam P McGuire, Binh An Nguyen Howard, Thane M Erickson, Suzannah K Creech

**Affiliations:** 1 VISN 17 Center of Excellence for Research on Returning War Veterans Waco, TX United States; 2 Central Texas Veterans Health Care System Temple, TX United States; 3 Department of Psychology and Counseling The University of Texas at Tyler Tyler, TX United States; 4 Department of Clinical Psychology Seattle Pacific University Seattle, WA United States; 5 Department of Psychiatry and Behavioral Sciences Dell Medical School of the University of Texas Austin, TX United States

**Keywords:** moral elevation, web-based intervention, pilot study, veterans, posttraumatic stress disorder, PTSD, moral injury

## Abstract

**Background:**

Veterans with posttraumatic stress disorder (PTSD) and moral injury can encounter several barriers to treatment, including limited access to care and low engagement with therapy. Furthermore, most treatment approaches focus on alleviating distress rather than cultivating positive experiences that could facilitate trauma recovery. A potential way to address these issues is through moral elevation: feeling uplifted and inspired by others’ virtuous actions.

**Objective:**

This study aimed to examine the feasibility and acceptability of a novel, web-based moral elevation intervention for veterans with PTSD symptoms and moral injury distress (Moral Elevation Online Intervention for Veterans Experiencing Distress Related to PTSD and Moral Injury [MOVED]). This mixed methods study also examined potential changes in PTSD symptoms, moral injury distress, quality of life, and prosocial behavior.

**Methods:**

In this pilot trial, 48 participants were randomized to a MOVED or control condition (24 participants per condition). Both conditions included 8 sessions and lasted 1 month. The MOVED intervention and all survey components across both conditions were administered online. Participants completed self-report measures that assessed PTSD symptoms, moral injury distress, quality of life, and prosocial behavior at baseline and follow-up. Veterans in the MOVED condition also completed individual qualitative interviews at follow-up. We coded qualitative responses to interviews and identified emergent themes.

**Results:**

Findings suggest the MOVED intervention was largely feasible, with evidence for moderate-to-high levels of participation, engagement, and retention in MOVED sessions. Both quantitative and qualitative results suggest veterans found MOVED to be acceptable and satisfactory at the overall treatment level. Furthermore, participants reported high scores for helpfulness and engagement at the session level. Veterans who completed MOVED reported large within-person decreases in PTSD symptoms (Cohen *d*=1.44), approximately twice that of veterans in the control condition (Cohen *d*=0.78). Those in MOVED also reported medium-sized increases in physical (Cohen *d*=0.71) and psychological domains of quality of life (Cohen *d*=0.74), compared with no meaningful changes in the control condition. Unexpectedly, MOVED veterans reported no decrease in moral injury distress, whereas veterans in the control condition endorsed a medium-sized decrease in the total score. There were no changes in prosociality for either condition. Qualitative feedback further supported high levels of perceived acceptability and satisfaction and positive treatment outcomes across a range of domains, including behaviors, cognitions, emotions, and social functioning. Veterans also recommended adaptations to enhance engagement and maximize the impact of intervention content.

**Conclusions:**

Overall, findings indicate that veterans with PTSD and moral injury distress were interested in an intervention based on exposure to and engagement with experiences of moral elevation. After further research and refinement guided by future trials, veterans may benefit from this novel approach, which may enhance treatment outcomes and increase treatment accessibility for those in need of additional trauma-focused care.

## Introduction

### Background

Veterans are at increased risk for exposure to traumatic events and are, therefore, at risk for major mental health consequences, such as posttraumatic stress disorder (PTSD) and moral injury [1,2]. PTSD is characterized by 4 core symptom clusters following a traumatic event: re-experiencing or intrusion, avoidance, changes in cognitions and mood, and arousal symptoms [3]. Moral injury occurs when a person witnesses or performs an act that violates their deeply held values and is associated with clinically significant levels of guilt, shame, and anger [4]. PTSD and moral injury are prevalent concerns for post-9/11 veterans in particular [5,6], and treatment for both is a high priority within this population, given their associations with poor mental health outcomes, functional impairment, and decreased social engagement [7-9].

Although addressing PTSD and moral injury is a priority, there are several barriers to successful treatment. Barriers include difficulties with treatment retention and the existence of residual symptoms after treatment [10,11], along with the limited availability of treatments that target both PTSD and moral injury [12,13]. To compound matters, many veterans who could benefit from mental health services do not engage in treatment [14]. Low use rates can be attributed, in part, to increased stigma around seeking mental health care [15] and accessibility issues that make it difficult to complete trauma-focused treatments [16], which typically involve weekly in-person psychotherapy sessions. Therefore, there is a need for novel approaches to overcome some of the existing barriers to increase treatment accessibility for this population.

### Moral Elevation and Theoretical Framework

Targeting moral elevation may provide one such novel approach to addressing PTSD and moral injury. Moral elevation (hereafter, “elevation”) is a positive emotion characterized by feeling inspired or moved after witnessing another person engage in a virtuous behavior (eg, an incredible act of compassion [17]). Elevation also involves physiological sensations—such as warmth in the chest, piloerection (goosebumps), or lump in the throat—and elicits a strong desire to imitate the witnessed virtue (eg, “I want to act compassionately too” [18]). Previous work posited elevation as a novel means for improving psychological health in veterans with PTSD and moral injury because its distinct features and subsequent psychosocial benefits are particularly antithetical to the features of trauma-related symptoms [19]. For example, eliciting elevation and increasing veterans’ tendency to be aware of others’ virtuous behaviors (ie, recognizing the goodness in others) may aid in reducing strong negative beliefs about others associated with PTSD (eg, “You cannot trust other people” [20]). Furthermore, because many veterans with PTSD and moral injury report difficulty experiencing positive feelings [21,22], repeated exposure to positive emotions such as elevation may help target the numbing features of trauma distress. Moreover, elevation’s action tendency could also motivate veterans to engage in behaviors that require greater social interaction (eg, acting compassionately toward another and prosocial behavior), which would counteract their social isolation tendencies—consistent with previous findings on the social benefits of elevation [23]. Within this theoretical framework, eliciting elevation as an intervention is distinct from typical trauma-focused treatments insofar as the primary objective is to instill strong, positive experiences, which is in contrast to other treatments that explicitly direct attention toward reducing symptoms. Consistent with other positive psychological approaches, this innovative path may present a unique opportunity to address gaps in facilitating trauma recovery for veterans with PTSD and moral injury.

### Support for Using Moral Elevation as a Therapeutic Tool

Broadly, past researchers have identified a series of benefits associated with elevation in nonclinical civilian populations, including increased prosocial behaviors, compassion, positive affect, and greater affiliation with others [24-26]. Although most studies have focused on the general population, several studies suggested that elevation might also be relevant for veterans exposed to traumatic events. First, an experimental study tested whether elevation can be induced in veterans with probable PTSD, which is an important question, given that many PTSD symptoms could presumably interfere with one’s ability to engage with this emotion (eg, numbing positive affect and having strong negative views of the world or others). The findings confirmed that veterans were able to experience elevation when exposed to video clips of others’ virtuous acts despite endorsing PTSD symptoms [27]. Furthermore, qualitative reports of positive cognitive, emotional, and motive-based reactions were consistent with the proposed theoretical framework and suggested possible relevance to trauma recovery. Using the same experimental design of eliciting elevation in veterans with video clips, another study linked higher levels of elevation with medium-sized decreases in shame, negative views of the self, and negative views of others [28]. Additional research also found that a higher level of naturally occurring elevation was associated with higher treatment engagement and lower posttreatment avoidance among veterans enrolled in a residential PTSD program [19]. Finally, in a civilian sample of people reporting subclinical PTSD symptoms following a campus shooting, higher elevation in the aftermath of the trauma prospectively predicted greater posttraumatic growth 4 months later [29].

Despite initial support from naturalistic observations and experimental studies, elevation has yet to be tested as a structured treatment for veterans with trauma-related distress. As a potential therapeutic tool, elevation may be particularly well suited for use in a web-based intervention, given prior evidence of the capacity to elicit this emotion using web-based induction methods (eg, video clips [24,30]). Research on the implementation of web-based positive psychology exercises has also demonstrated high levels of feasibility, acceptability, and positive outcomes [31,32]. Given the need to increase the accessibility and decrease the barriers to trauma-focused care for this population, an elevation intervention completed online could facilitate treatment engagement among veterans who are unable to access initial treatment because of availability or cost as well as veterans who may be averse to initiating in-person treatment.

### This Study

In this mixed methods study, we developed and tested a novel web-based elevation intervention titled *Moral Elevation Online Intervention for Veterans Experiencing Distress Related to PTSD and Moral Injury (MOVED)*. Consistent with the Obesity-Related Behavioral Intervention Trials model for developing behavioral treatments for chronic diseases [33], this pre-efficacy pilot trial was designed to examine the overall feasibility and acceptability of MOVED for veterans with PTSD and moral injury distress. This study included 4 exploratory aims that used quantitative and qualitative data. First, we assessed the feasibility of randomization and completion of 2 conditions—a MOVED treatment condition and a control condition. Second, we examined whether MOVED elicited elevation as intended. Third, we assessed the acceptability of and satisfaction with MOVED. Finally, we examined potential changes in the targeted outcomes of PTSD symptoms, moral injury distress, quality of life, and prosocial behavior.

## Methods

### Participants

The researchers mailed recruitment letters to veterans of the recent US wars in and around Iraq and Afghanistan, who were enrolled in a regional Veteran Affairs Healthcare System and received at least 2 PTSD diagnoses on record in the past 6 months (indicating some continuation of having met diagnostic criteria). Study staff followed up the recruitment letters with up to 3 phone calls per veteran to inquire about potential interest.

Interested veterans completed a phone screen to determine initial eligibility based on the following inclusion criteria: (1) aged at least 18 years, (2) US Iraq or Afghanistan war veteran, (3) English speaking, (4) internet access, (5) significant PTSD symptoms (≥33 on the Posttraumatic Checklist for Diagnostic and Statistical Manual of Mental Disorders, Fifth Edition [PCL-5] [34,35]), (6) elevated levels of distress about a potentially morally injurious event on the Moral Injury Events Scale (eg, slightly, moderately, or strongly agreeing with feeling “troubled” by at least 1 event [36]), and (7) willingness to identify a *significant other* (eg, friend, partner or spouse, or coworker) who interacted with the veteran at least weekly and would complete pre-post observer ratings of the veteran’s behavior.

After fulfilling the initial eligibility criteria, veterans provided consent and completed a baseline assessment over the phone. Trained staff administered a brief clinical interview (Mini-International Neuropsychiatric Interview [MINI] [37]) and self-report measures of the main study variables. Final eligibility for randomization was determined based on the following exclusion criteria: (1) active psychosis (MINI), (2) current substance or alcohol use disorder active in the past 3 months (MINI), (3) history of severe traumatic brain injury indicated by the Ohio State University Traumatic Brain Injury Identification Method [38], and (4) current suicide risk score of ≥2 on the suicide item of the Beck Depression Inventory-II [39]. Given the early phase of treatment development and our emphasis on assessing feasibility and acceptability, veterans were not excluded if they were currently enrolled in other treatments.

**Figure 1:**
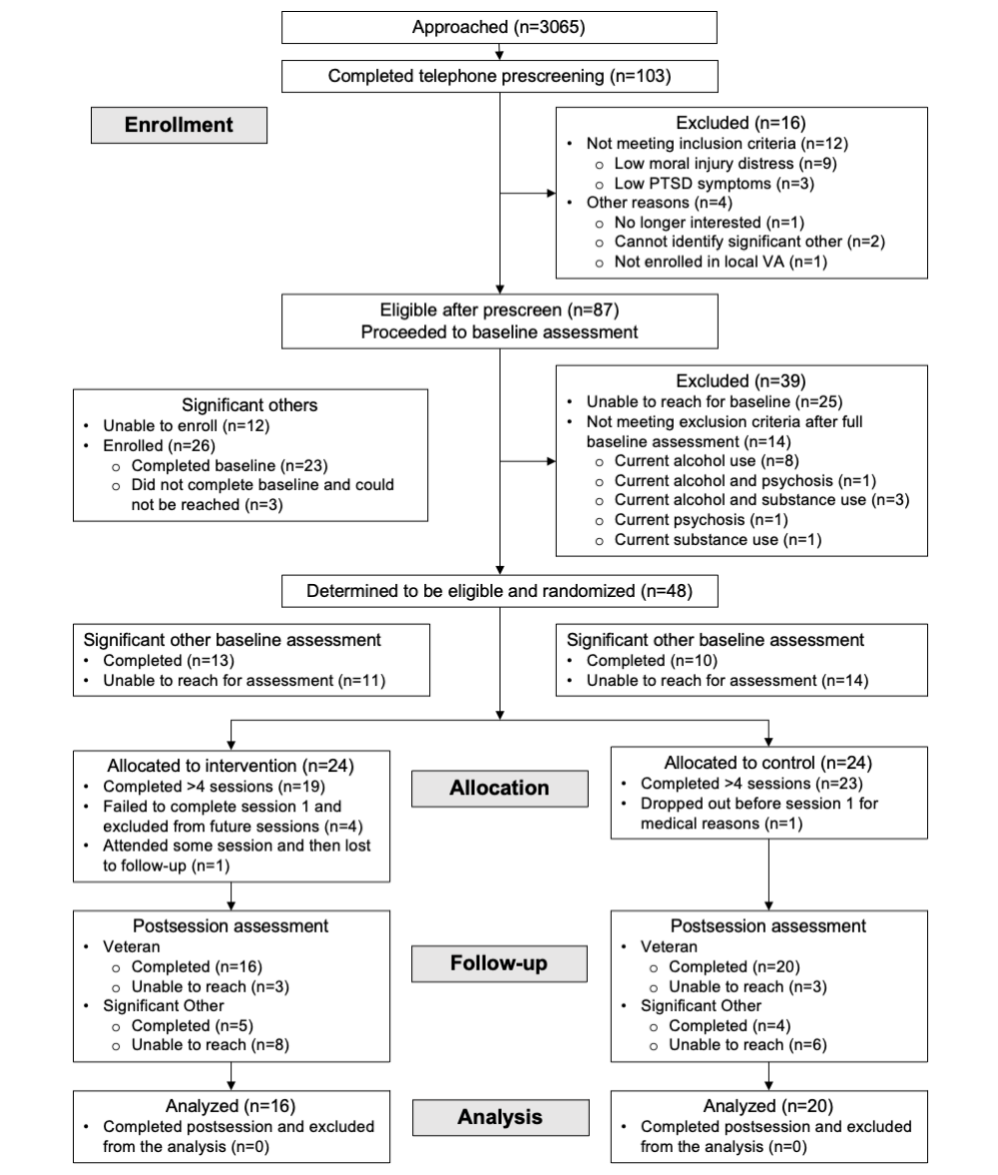
CONSORT (Consolidated Standards of Reporting Trials) diagram. PTSD: posttraumatic stress disorder; VA: Veterans Affairs.

After the baseline assessment, we randomized eligible veterans to a treatment (MOVED) or control condition using a random sequence generator (1:1 allocation; Figure 1). Study staff remained blind to the condition until participants received notification of the assigned condition. The MOVED condition included 8 web-based sessions consisting of elevation intervention content (refer to the subsequent section) and repeated surveys of mood and social factors experienced in the past few days. The control condition also included 8 web-based sessions but was limited to completing the same repeated surveys and involved no intervention content or exercises. Participants in the control condition were instructed to simply complete 8 repeated surveys at designated times across 4 weeks. The final sample included 48 veterans. Treatment (n=24) and control (n=24) condition participants did not differ significantly in demographic and military characteristics (Table 1).

**Table 1:** Demographic characteristics of the pilot sample^a^.

Variable				Treatment condition (n=23)^b^		Control condition (n=24)		Test statistic							All participants (n=47)^b^
								*t* test *(df)*		chi-square (*df*)		*P* value			
Age (years), mean (SD)				43.42 (6.45)		45.38 (8.85)		0.88 (42)				.39		44.40 (7.72)	
**Gender, n (%)**										0.01 (1)		.94			
	Man		19 (79)		20 (83)								39 (81)		
	Woman		5 (21)		4 (17)								9 (19)		
**Race, n (%)**										11.54 (11)		.40			
	American Indian or Alaska Native		1 (4)		1 (4)								2 (4)		
	Asian or Asian American		1 (4)		0 (0)								1 (2)		
	Black or African American		10 (43)		9 (38)								19 (44)		
	Native Hawaiian or Pacific Islander		1 (4)		1 (4)								2 (4)		
	Other		1 (4)		2 (8)								3 (6)		
	Unknown		1 (4)		0 (0)								1 (2)		
	White		7 (30)		13 (54)								20 (43)		
Ethnicity (Hispanic), n (%)				6 (26)		2 (8)				1.35 (1)		.25		8 (17)	
Education years, mean (SD)				14.09 (2.50)		14.88 (2.15)		1.16 (43)				.25		14.49 (2.34)	
**Education degree, n (%)**										4.84 (6)		.57			
	High school or GED^c^		4 (17)		2 (8)								6 (13)		
	Technical school certification		0 (0)		2 (8)								2 (4)		
	Some college		8 (35)		5 (21)								13 (28)		
	College graduate degree		8 (35)		10 (42)								18 (38)		
	Postgraduate degree		3 (13)		5 (21)								8 (17)		
**Relationship status, n (%)**										7.46 (8)		.49			
	Married		14 (60)		16 (67)								30 (64)		
	Divorced		3 (13)		1 (4)								4 (9)		
	Widowed		1 (4)		0 (0)								1 (2)		
	Single, in a relationship		3 (13)		5 (21)								8 (17)		
	Single, no relationship		2 (9)		2 (8)								4 (9)		
**Income (US $), n (%)**										4.58 (6)		.60			
	0-14,999		1 (4)		1 (4)								2 (4)		
	15,000-29,999		3 (13)		3 (12)								6 (13)		
	30,000-44,999		5 (22)		5 (21)								10 (21)		
	45,000-59,999		3 (13)		7 (29)								10 (21)		
	60,000-74,999		5 (22)		5 (21)								10 (21)		
	75,000-89,999		5 (22)		1 (4)								6 (13)		
	≥90,000		1 (4)		2 (8)								3 (6)		
**Military branch, n (%)**										5.44 (7)		.61			
	Army		20 (87)		21 (88)								41 (87)		
	Marine Corps		3 (13)		1 (4)								4 (9)		
	Air Force		2 (9)		1 (4)								3 (6)		
	Navy		0 (0)		2 (8)								2 (4)		
**Military rank, n (%)**										1.69 (4)		.79			
	Enlisted E1-E4^d^		6 (26)		8 (33)								14 (30)		
	Enlisted E5-E6		9 (39)		9 (38)								18 (38)		
	Enlisted E7-E9		6 (26)		5 (21)								11 (23)		
	O4-O9^e^		2 (9)		1 (4)								3 (6)		
	WO1-WO5^f^		0 (0)		1 (4)								1 (2)		
Combat exposure (yes), n (%)				23 (100)		21 (88)				1.34 (1)		.25		44 (94)	
Deployment number, mean (SD)				2.35 (1.61)		3.04 (1.81)		1.39 (45)				.17		2.70 (1.73)	
**Deployment theater, n (%)**										5.76 (6)		.45			
	Iraq		22 (96)		20 (83)								42 (89)		
	Afghanistan		5 (22)		8 (33)								13 (28)		
	**Other**		7 (30)		9 (38)								16 (34)		
		Kuwait	2 (9)		3 (12)								5 (11)		
		Somalia	0 (0)		1 (4)								1 (2)		
		Kosovo	1 (4)		1 (4)								2 (4)		
		Cuba	0 (0)		1 (4)								1 (2)		
		Egypt	2 (9)		0 (0)								2 (4)		
		Korea	1 (4)		0 (0)								1 (2)		
		Honduras	1 (4)		0 (0)								1 (2)		
		Not specified	0 (0)		3 (12)								3 (6)		

^a^Participants were allowed to select multiple choices for several categories, including race, military branch, and deployment theaters; thus, the sum counts for these variables may be >24 within each condition.

^b^Demographic data for the treatment condition are limited to 23 participants because data were missing for 1 participant.

^c^GED: General Educational Development.

^d^E: enlisted.

^e^O: commissioned officers.

^f^WO: warrant officers.

### Procedure

After randomization, study staff contacted significant others using the information provided by veteran participants. Study staff attempted to contact significant others using up to 3 phone calls each and asked the associated veteran participants to notify their significant others that the staff members were attempting to contact them. Significant other candidates were screened for the following inclusion criteria: (1) aged ≥18 years, (2) spoke English, (3) had internet access, (4) was willing to complete web-based surveys at pre-post sessions, and (5) reported interacting with the associated veteran at least weekly. Significant others provided consent to participate.

Across both conditions, participants completed 8 self-administered sessions: 2 sessions per week for 4 weeks. The sessions were completed online independently and did not involve a therapist or coach. Every Monday and Thursday, participants received an email at 6 AM with a link to the assigned session. For the control condition, the link directed participants to a session that was composed only of repeated surveys. For the MOVED condition, the link directed participants to a session that included the same surveys, followed by the intervention content. Participants who did not complete the session by 6 PM received a reminder email, and those who did not complete the session that day received a reminder call the next morning, encouraging them to complete the session within the next 12 hours. After 4 weeks of sessions, all participants, including veterans and significant others across both conditions, received an email link to a follow-up assessment; veteran participants in the treatment condition subsequently engaged in a 1-hour recorded qualitative interview. Staff sent follow-up emails and provided up to 3 phone call reminders to complete the follow-up assessment (ie, postsession assessment) and interview. This method was also used to reinitiate contact with participants who dropped out or failed to complete sessions after enrollment and randomization. All surveys—baseline, repeated session, and postsession—were administered online via Qualtrics (Qualtrics International Inc).

### Ethics Approval

The Central Texas Veterans Health Care System institutional review board approved all study procedures (study protocol #00702). First, all veterans and significant others were mailed a consent document that summarized their study participation. Next, a member of the study staff reviewed that document with participants over the phone, and then participants provided verbal consent. Participants received US $30 for the baseline assessment and US $15 for completing each web-based survey session (a total of 8 sessions). Participants in the control condition received US $30 for the postsession assessment, whereas those in the intervention condition received US $40 for the postsession assessment and interview (maximum compensation: US $180 and US $190, respectively). Significant others received a gift card worth US $20 for each baseline and postsession assessment (maximum of US $40).

### Intervention Content

#### Overview

Every session in MOVED had 3 core steps to the intervention delivery: elevation induction, reflection, and goal setting, as described in the subsequent section. Before establishing these components, the first session started with psychoeducation: orientation to the intervention and its features; the definition of elevation as a positive emotion; an overview of virtues that one might recognize as elicitors of elevation; and definitions for the virtues of courage, forgiveness, gratitude, hope, love, perseverance, and selfless service. In this intervention, virtues were described as *strengths* in an effort to use language that might be perceived as similar to and consistent with military culture. The full intervention manual is available on the Open Science Framework (OSF) page [40].

#### Elevation Induction

First, each MOVED session induced an elevation response by exposing participants to stimuli depicting others engaging in a virtuous action or a remarkable display of character strength. Induction methods included watching a video clip depicting this content (first 4 sessions) or *cued recall* by reflecting on a personal memory of witnessing a virtuous behavior (last 4 sessions)—methods from extant elevation research [24,30]. Previous work suggests that mixing these induction methods reduces the likelihood of habituation to elevating stimuli [24]. Video inductions preceded recall inductions to facilitate practice in recognizing virtuous behaviors in a structured context before identifying virtuous behaviors from their lives.

#### Reflection Exercise

After each induction, veterans were asked to briefly reflect on the behavior witnessed or recalled by journaling about their reactions. Specifically, they provided typed responses to a set of prompts that aimed to facilitate attention to the goodness of others, the capacity to experience positive emotions, and motivations to engage in potentially helpful behavior (eg, imitating virtuous behaviors, self-improvement, and connecting with others).

#### Goal Setting

Finally, based on experiments incorporating goal setting into elevation interventions [24], veterans set a goal to be completed in the next few days before the following session. For the first 2 sessions, the goal was preselected to make adherence easier and to orient participants to completing goals in response to elevation inductions (Table 2). In other sessions, veterans were instructed to set their own unique goal that “a) you feel motivated to do after thinking about the video; b) is related to something important to you or aligns with your personal values; and c) could be realistically completed before the next session.” Next, veterans were asked to verify that their self-generated goals met the criteria for a Specific, Measurable, Achievable, Relevant, and Timely goal. At the beginning of each session, veterans checked in about their most recent goal and reported on goal completion.

**Table 2:** Schedule and content of MOVED sessions.

Session number (day of week)	Elevation induction	Goal
1 (Monday)	Video 1: bus driver cooks and delivers food to the homeless	Tell someone about the video
2 (Thursday)	Video 2: father and son compete in marathons and triathlons	Tell someone about the video and describe the witnessed strengths and personal reaction
3 (Monday)	Video 3: former child soldier disarms land mines	Self-generated SMART^a^ goal
4 (Thursday)	Video 4: veteran provides tiny homes to help other veterans	Self-generated SMART goal
5 (Monday)	Recall exercise	Self-generated SMART goal
6 (Thursday)	Recall exercise	Self-generated SMART goal
7 (Monday)	Recall exercise	Self-generated SMART goal
8 (Thursday)	Recall exercise	Identify long-term goal for after program and describe what strengths *move* you and where you can search those out in daily life

^a^SMART: Specific, Measurable, Achievable, Relevant, and Timely.

### Measures

#### Baseline and Postsession Measures

##### Demographic and Military History

At baseline, participants reported their demographic characteristics and military history, including combat exposure, deployment locations, and number of deployments.

##### PTSD Symptoms

The PCL-5 assessed the severity of PTSD symptoms in the past *month* during the initial screen and in the past *week* at the postsession assessment [35]. Participants rated 20 items on a Likert scale ranging from 0 (*not at all*) to 4 (*extremely*). Total scores were calculated by summing all items, with higher scores indicating greater severity. The PCL-5 has demonstrated reliability and validity in previous studies, with scores of ≥33 associated with a probable PTSD diagnosis [34,41]. Internal consistency in the present sample was α=.84 (95% CI 0.76-0.92) at baseline and α=.94 (95% CI 0.91-0.97) at the postsession assessment.

##### Moral Injury–Related Distress

The Expressions of Moral Injury Scale includes 17 items, which assessed distress related to moral injury on a scale ranging from 1 (*strongly disagree*) to 5 (*strongly agree*) [42]. The items were summed to create a total score and 2 subscale scores: self-focused and other-focused expressions of moral injury. Higher scores indicated greater moral injury-related distress. In this study, internal consistency for the total score was α=.92 (95% CI 0.89-0.95) at baseline and α=.93 (95% CI 0.89-0.97) at the postsession assessment.

##### Quality of Life

The World Health Organization Quality of Life-BREF is an abbreviated 26-item version of the World Health Organization Quality of Life-100 assessment, which assessed quality of life domains (physical, psychological, social, and environmental) on a 5-point scale [43]. Summing items within each domain yields subscales, with higher scores indicating higher quality of life. In our study, α ranged from .75 to .82 at baseline and from .83 to .89 at the postsession assessment.

##### Prosocial Behavior

The Prosocialness Scale assessed prosocial behavior as rated by both the veteran and significant other (observer ratings) [44]. Participants rated 16 items on a scale ranging from 1 (*never/almost never true*) to 5 (*almost always/always true*), with higher summed scores indicating more prosocial behaviors. In the present sample, internal consistency was α=.93 (95% CI 0.90-0.95) at baseline and α=.95 (95% CI 0.92-0.97) at the postsession assessment.

#### Repeated Session Measures

##### State Elevation

At the time of the study, no standardized measures existed for assessing state elevation. Therefore, 12 items used in previous studies [28,45,46] were administered immediately after completing the elevation induction task at each session to assess the state elevation response. Participants rated each item on a scale ranging from 0 (*not at all*) to 4 (*extremely*), with higher summed totals representing greater state elevation.

##### Exercise-Specific Satisfaction

Four items assessed the perceived satisfaction with and helpfulness of the exercises. Items were rated on a scale ranging from 0 (*not at all*) to 8 (*extremely*) and administered at the end of each session.

The state elevation and exercise-specific satisfaction measures were administered only to veterans in the treatment condition because these measures assessed experiences with the intervention content, which were exclusive to the MOVED condition and thus irrelevant to the control condition. A set of additional repeated measures was administered to both the treatment and control conditions to assess affect, behaviors, and social interactions between the sessions. The results of these measures are not reported here, but a detailed description of the measures and full study procedures can be found on the OSF project page [40].

#### Posttreatment Program Evaluation

##### Acceptability and Satisfaction

An adaptation of the Treatment Evaluation Inventory-Short Form (TEI-SF) assessed the acceptability of the intervention, with specific reference to its acceptability as an intervention for targeting PTSD and moral injury [47]. Seven items were rated on a Likert scale ranging from 1 (*strongly disagree*) to 5 (*strongly agree*). Item scores were evaluated individually, rather than using a sum score.

The 8-item Client Satisfaction Questionnaire-8 (CSQ-8) assessed the overall satisfaction with the intervention [48]. Items were rated on a scale ranging from 1 to 4, with higher summed scores indicating greater satisfaction. In this study, α=.95 (95% CI 0.92-0.97).

##### Qualitative Interview

Participants randomized to the treatment condition completed a 1-hour semistructured interview that included questions regarding initiation or enrollment into the treatment, engagement, session and treatment experiences, and suggested revisions. The interview guide is available on the OSF page.

### Data Analytic Plan

All data management and analyses were conducted using the R software (R Foundation for Statistical Computing) [49]. To assess the feasibility and acceptability of MOVED, we calculated descriptive statistics for recruitment and retention data, self-report measures that focused on evaluating the components of the intervention, and state elevation scores. For all evaluation measures, the midpoint score for a given measure was used as an indicator of adequate feasibility and acceptability (eg, average or medium level of acceptability or higher). The midpoint score for state elevation (2) was also used as an indicator of the feasibility of eliciting a moderate level of elevation.

Qualitative interviews were transcribed and then coded using an inductive approach by 2 members of the research team and the first author. The 2 coders reviewed all transcriptions independently, identified codes based on interview responses, and then met to discuss and modify the codes. A consensus codebook was established and reapplied to all transcriptions. Emergent themes and subthemes were identified by the first author. The final codebook and list of identified themes are available on the OSF page.

Preliminary examination of outcome measures was conducted via paired samples *t* tests (2-tailed) with the *base stats* package (*t.test*) and Cohen *d* effect sizes for each condition with the *rstatix* package [50]. We used the *tidyverse* package [51] to produce all the figures. The assessment of changes in pre-post measures included all participants who were randomized and provided postsession data.

## Results

### Enrollment and Randomization

Among those who completed a telephone screen, 84.5% (87/103) were eligible for further assessment. Among those who completed the baseline appointment, 55% (48/87) met full eligibility and were randomized into either the treatment (24/48, 50%) or control condition (24/48, 50%). Figure 1 presents a CONSORT (Consolidated Standards of Reporting Trials) diagram. Regarding significant other participants, only 54% (26/48) of those contacted completed the baseline observer rating.

In the treatment condition, participants were required to complete session 1 before being allowed to proceed to future sessions because the orientation and psychoeducation provided in session 1 were considered essential to successful engagement in the remaining sessions. A total of 17% (4/24) of participants did not complete session 1 despite repeated contact attempts; therefore, they were excluded from future analysis. In the control condition, of 24 participants, 1 (4%) participant dropped out after randomization but before session 1 for medical reasons.

The qualitative interview results highlighted several reasons why participants decided to enroll and potentially participate in MOVED. Veterans mostly described reasons that included a desire to address mental health symptoms, improve general life functioning, and improve social functioning, particularly with family members. For example, “I’m willing to do whatever because my wife you know, she’s hurting. She’s struggling [and] you know, I’m struggling and I want help.” Several participants also indicated that the telehealth component contributed to their decision to enroll.

### Engagement and Completing Sessions

Out of the 8 possible sessions, veterans randomized to the MOVED condition completed an average of 6 (mean 5.96, SD 3.18) sessions, and those randomized to the control condition completed an average of 7 (mean 7.08, SD 1.74) sessions; 63% (15/24) completed all 8 sessions in the treatment condition. The qualitative results indicated several strengths of the MOVED format that contributed to its high feasibility and engagement. Specifically, many veterans described the use of videos within sessions, self-administered format, and flexibility of completing sessions on their own as strengths. For example, “I am glad I had the opportunity to do it on my own time or when I had the time...being flexible with time was very good.” In addition, some reported enhanced openness and not fearing judgment because of the web-based format:

The online interaction made it a little easier to be open and honest about things going on, and how I feel about them; It gives you more of a chance to honestly assess yourself without feeling judged.

We also examined the feasibility of the goal-setting exercise by examining descriptive statistics for the number of weeks participants reported completing the previous session’s goal. On average, participants completed 4.32 (range 0-7) of 7 session goals. Notably, the highest rate of completion was found in goals from sessions 1 (15/18, 83.33%) and 2 (14/18, 77.78%), that is, the predetermined goals assigned to veterans rather than the participant-generated goals.

Although the number of goals completed by participants was more than the number of incomplete goals, the qualitative responses suggested that the experience of setting and completing goals was difficult for some participants. Several veterans noted difficulties in remembering their goals, experiencing difficulties in completing goals because of low motivation and PTSD symptoms, and challenges with balancing goal-oriented activities with other responsibilities or personal or work schedules.

### Follow-up Analysis

Among the 48 randomized participants, 36 (75%) completed the postsession survey: 16 (67%) out of 24 and 20 (83%) out of 24 from the treatment and control conditions, respectively. All participants who completed the postsession survey were included in subsequent analyses. Only 9 significant others completed the postsession survey: 19% of the desired sample size (N=48) and 39% of those who completed the baseline survey (n=23). Given the low response rate for significant other participants at follow-up, we did not examine change scores in observer ratings.

### Eliciting Elevation

**Figure 2:**
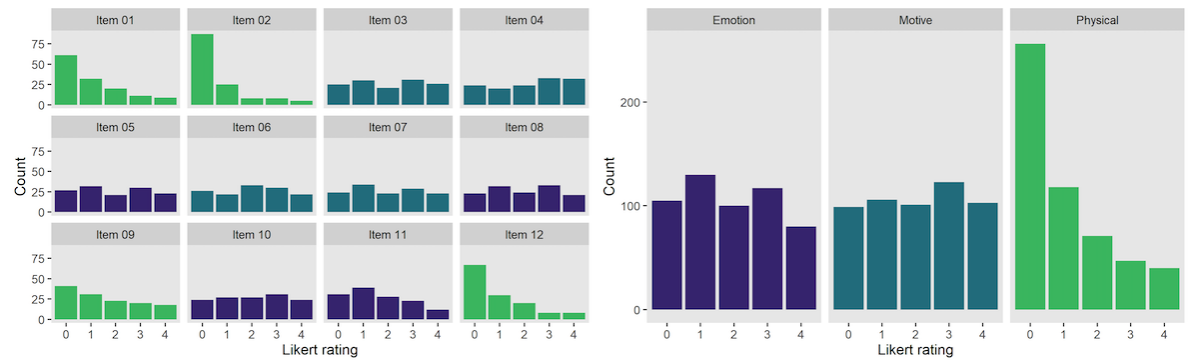
Distribution of the state elevation scores at the item and domain levels, aggregated across all participants and sessions.

For participants in the treatment condition, we examined descriptive statistics for self-reported state elevation to determine whether the induction exercises elicited elevation as intended. The mean score when all 12 items were included, aggregated across all sessions, was 1.63 (SD 1.12), with a median of 1.58 and a range of 0 to 4. Because the mean was below the midpoint score of 2—lower than expected for sessions designed to elicit elevation—we further inspected elevation scores by examining the distribution of endorsed items. Notably, several items were positively skewed, and most items did not appear to be normally distributed in this small sample (Figure 2). When items were sorted into the targeted domains of emotional reaction, physical reaction, and motives, positive skewness was particularly problematic for physical reaction items (eg, goosebumps, choked up, and warmth in the chest), with most participants clustering around no physical response to elevation stimuli. Physical items had a low average score (mean 1.03, SD 1.07; median 0.75), whereas scores were closer to the midpoint for emotion (mean 1.85, SD 1.24; median 2) and motive items (mean 2.01, SD 1.29; median 2).

Given the low response (and skew) with physical items, we recalculated the total state elevation scores after excluding the scores for physical items. The resulting 8-item mean score was 1.93 (SD 1.23; median 2). Further breakdown of the state elevation scores by session and session type is available on the OSF page.

Accordingly, veterans’ qualitative responses suggested mixed results regarding elevation responses to the videos and recall exercises. Several veterans described the exercises as helpful and reported experiencing positive emotions—consistent with the intended purpose of the induction exercises—such as feeling inspired and motivated in response to the exercises. With regard to the videos, in particular, veterans articulated the recognition of a wide range of virtues, including commitment, compassion, love, and selflessness—suggesting an awareness of elicitors that lead one to experience elevation. For instance, “He did it all out of the goodness of his heart,” and “Just watching that and seeing how the father just loved his son so much he was willing to put himself through these marathons, just to see his son smile.” Veterans also described motives consistent with the theoretical framework, including a desire to help and connect with others. For example, “It gave me the knowledge and the confidence to say, ‘You know what, I can help somebody else, and help them a lot.’”

However, several veterans also described barriers to experiencing a positive response during the induction exercises. Regarding the videos, veterans with little to no positive responses indicated that some of the videos were not relatable, “cheesy,” or in some cases, elicited a negative response. For example, negative responses included being reminded of social disconnection, being reminded of a traumatic event, or endorsing negative judgments about the people featured in the video. Veterans also described challenges with identifying events that elicited elevation in their own life with respect to the recall exercises, particularly in the context of the COVID-19 pandemic:

Because interpersonal communication is now very limited or is still non-existent for some, people are still not really going out and interacting with other people and having conversations because of what is going on.

### Acceptability and Satisfaction

For the overall treatment, the mean and median scores across both the TEI-SF and CSQ-8 items were above the item range midpoint (TEI-SF=3; CSQ-8=2.5). These results suggest that veterans found the overall intervention acceptable and satisfactory (Table 3).

Regarding satisfaction with individual sessions and their content as assessed by the exercise-specific satisfaction measure, mean and median scores for items assessed at each session were above the item range midpoint (4 out of 0 to 8). Specifically, veterans indicated that the sessions were moderately helpful (mean 4.95, SD 2.37; median 6.00), that they moderately benefited from a session’s elevation exercise (mean 4.64, SD 2.69; median 5.00), that the goal-setting assignment was moderately helpful (mean 5.11, SD 2.59; median 6.00), and that they were highly engaged with the session (mean 6.22, SD 1.87; median 7.00). The scores were relatively similar across sessions and session types (Table 4).

Themes from the qualitative data further supported high levels of perceived acceptability and satisfaction. Veterans reported feeling comfortable with MOVED, described it as helpful, and stated that they looked forward to the sessions. Some veterans noted that they saw MOVED as a good first step in the process of recovering from trauma exposure, and several veterans mentioned that they explicitly endorsed the intervention when speaking to other veterans. Regarding the specific structure, many veterans reported being satisfied with features such as the session length, the web-based format, and having 2 sessions each week and highly endorsed the use of videos (eg, “I think twice a week was good. I actually looked forward to it every week.”).

**Table 3:** Descriptive statistics for postsession acceptability and satisfaction measures^a^.

Variables and items		Value	
		Mean (SD)	Median (range)
**Treatment Evaluation Inventory-Short Form**			
	1. I find this intervention to be an acceptable way of dealing with problems related to PTSD^b^ and moral injury.	3.62 (1.01)	4.0 (2-5)
	2. I would be willing to use this procedure if I wanted to seek help again.	4.00 (0.95)	4.0 (2-5)
	3. I like the procedures used in this intervention.	3.94 (0.91)	4.0 (2-5)
	4. I believe this intervention is likely to be effective.	3.69 (0.93)	4.0 (2-5)
	5. I believe others would experience discomfort during this intervention.^c^	2.75 (0.92)	3.0 (1-4)
	6. I believe this intervention is likely to result in permanent improvement.	3.25 (0.67)	3.0 (2-4)
	7. Overall, I have a positive reaction to this intervention.	3.94 (0.84)	4.0 (2-5)
**CSQ-8^d^**			
	1. Quality of service received	3.44 (0.72)	4.0 (2-4)
	2. Get service you wanted	3.31 (0.78)	3.5 (2-4)
	3. Extent program met your needs	2.88 (0.94)	3.0 (1-4)
	4. Recommend program to friend in need of similar help	3.19 (0.74)	3.0 (2-4)
	5. Satisfied with amount of help received	2.94 (0.76)	3.0 (2-4)
	6. Services received helped deal more effectively with problems	2.88 (0.79)	3.0 (1-4)
	7. Overall, satisfied with service received	2.94 (0.76)	3.0 (2-4)
	8. If you were seeking help again, would you come back to the program?	3.25 (0.76)	3.0 (2-4)
	CSQ-8 total	24.81 (5.26)	25.0 (15-32)

^a^Higher scores on the Client Satisfaction Questionnaire-8 indicate greater satisfaction (Likert scale anchors differ for each question). Higher scores on the Treatment Evaluation Inventory-Short Form indicate greater satisfaction (1=*Strongly disagree*, 2=*Disagree*, 3=*Neutral*, 4=*Agree*, and 5=*Strongly agree*).

^b^PTSD: posttraumatic stress disorder.

^c^Item 5 is negatively valenced, with lower scores representing greater satisfaction.

^d^CSQ-8: Client Satisfaction Questionnaire-8.

**Table 4:** Descriptive statistics for exercise-specific session acceptability and satisfaction items^a^.

Session		Item 1: rate how helpful this session was to you		Item 2: rate how much you benefitted from this session’s exercise		Item 3: rate how helpful the goal assignment was to you			Item 4: rate how much you were fully engaged with the material for this session	
		Mean (SD)	Median	Mean (SD)	Median	Mean (SD)	Median	Mean (SD)		Median
Total		4.95 (2.37)	6.0	4.64 (2.69)	5.0	5.11 (2.59)	6.0	6.22 (1.87)		7.0
**Video sessions**		4.81 (2.35)	5.0	4.64 (2.60)	5.0	5.13 (2.56)	6.0	6.43 (1.66)		7.0
	1	4.40 (2.52)	4.5	4.15 (2.78)	4.0	5.50 (2.76)	7.0	6.30 (1.98)		6.5
	2	5.13 (2.22)	6.0	4.56 (2.66)	6.0	4.75 (2.52)	6.0	6.44 (1.63)		6.5
	3	4.56 (2.17)	4.5	4.61 (2.50)	5.0	4.89 (2.70)	6.0	6.22 (1.66)		7.0
	4	5.33 (2.55)	6.0	5.40 (2.47)	6.0	5.33 (2.32)	6.0	6.87 (1.25)		7.0
**Recall sessions**		5.11 (2.39)	6.0	4.63 (2.82)	6.0	5.08 (2.64)	6.0	5.98 (2.07)		6.5
	5	5.24 (2.08)	5.0	4.88 (2.62)	5.0	5.12 (2.83)	6.0	6.65 (1.37)		7.0
	6	4.57 (2.34)	4.5	4.36 (2.92)	4.5	4.93 (2.53)	6.0	5.29 (2.33)		6.0
	7	4.82 (2.79)	6.0	4.24 (3.11)	4.0	4.71 (3.06)	6.0	5.76 (2.36)		6.0
	8	5.75 (2.35)	6.0	5.07 (2.79)	6.0	5.56 (2.19)	6.0	6.13 (2.06)		7.0

^a^Score for each item ranged from 0 to 8.

### Changes in Targeted Outcomes

Total PTSD symptoms and all 4 symptom clusters demonstrated significant pre-post decreases in both the treatment and control conditions (Table 5). However, participants in the treatment condition reported large within-person effects for overall symptoms (Cohen *d*=1.44) and each symptom cluster (Cohen *d* range 0.83-1.60), whereas those in the control condition reported medium-sized effects for overall symptoms (Cohen *d*=0.78) and clusters (Cohen *d* range 0.51-0.62). Notably, the average change score for the treatment condition fell within the range of a 15-point decrease to 18-point decrease on the PCL-5—a marker of reliable change in PTSD symptoms [52]. By contrast, moral injury-related distress remained largely unchanged in the treatment condition. Unexpectedly, in the control condition, there was a medium-sized, significant decrease in the overall moral injury distress (Cohen *d*=0.51) but smaller effect sizes with CIs near 0 for the 2 subscale scores of self- and other-focused moral injury.

Regarding quality of life, MOVED participants reported a medium, significant increase in quality of life in the physical (Cohen *d*=0.71) and psychological domains (Cohen *d*=0.74) but no changes in the social or environmental domains. Participants in the control condition reported no significant changes in any of the domains. Neither condition endorsed any changes in prosociality.

**Table 5:** Within-person effects for pre-post measures across conditions.

Variable			Treatment condition (n=16)									Control condition (n=20)						
			Mean difference		*t* test (*df*)		*P* value		Cohen *d* (95% CI)		Mean difference			*t* test (*df*)		*P* value		Cohen *d* (95% CI)
**PCL-5^a^ total^b^**			−*17.71*^c^		*5.78 (15)*		*<.001*		*1.44 (1.00 to 2.49)*		−*9.05*			*3.48 (19)*		*.002*		*0.78 (0.35 to 1.38)*
	PCL-5 re-experiencing	−*6.13*		*6.40 (15)*		*<.001*		*1.60 (1.08 to 2.74)*		−*2.50*			*2.79 (19)*		*.01*		*0.62 (0.20 to 1.23)*	
	PCL-5 avoidance	−*1.56*		*3.43 (15)*		*.004*		*0.86 (0.57 to 1.33)*		−*1.25*			*3.10 (19)*		*.006*		*0.69 (0.38 to 1.08)*	
	PCL-5 cognition and mood	−*5.69*		*4.77 (15)*		*<.001*		*1.19 (0.65 to 2.33)*		−*3.05*			*2.29 (19)*		*.03*		*0.51 (0.07 to 1.08)*	
	PCL-5 hyperarousal	−*4.38*		*3.33 (15)*		*.005*		*0.83 (0.52 to 1.29)*		−*2.23*			*2.71 (19)*		*.01*		*0.61 (0.20 to 1.07)*	
**EMIS^d^ total**			−0.20		0.06 (14)		.96		0.01 (−0.45 to 0.91)		−*3.65*			*2.30 (19)*		*.03*		*0.51 (0.08 to 1.25)*
	EMIS self	−1.20		0.70 (14)		.50		0.18 (−0.30 to 1.02)		−2.15			1.77 (19)		.09		0.40 (0.05 to 1.07)	
	EMIS other	1.00		0.39 (14)		.70		0.10 (−0.76 to 0.55)		−1.50			2.09 (19)		.05		0.47 (0.05 to 0.96)	
WHOQOL^e^ physical subscale			*1.37*		*2.76 (14)*		*.02*		*0.71 (0.32 to 1.31)*		0.03			0.08 (19)		.94		0.02 (−0.43 to 0.55)
WHOQOL psychological subscale			*1.22*		*2.87 (14)*		*.01*		*0.74 (0.26 to 1.55)*		0.37			1.14 (19)		.27		0.25 (−0.17 to 0.79)
WHOQOL social subscale			1.11		1.18 (11)		.26		0.34 (−0.27 to 1.02)		−0.07			0.16 (18)		.88		0.04 (−0.41 to 0.49)
WHOQOL environmental subscale			0.76		1.18 (14)		.26		0.30 (−0.18 to 0.92)		−0.06			0.14 (19)		.89		0.03 (−0.38 to 0.59)
Prosocial (veteran)			−1.27		0.30 (14)		.77		0.08 (−0.45 to 0.71)		0.05			0.03 (19)		.98		0.01 (−0.53 to 0.42)

^a^PCL-5: Posttraumatic Checklist for Diagnostic and Statistical Manual of Mental Disorders, Fifth Edition.

^b^One of the participants only completed the PCL-5 at baseline because of technical errors but completed all postsession measures; therefore, the PTSD symptom comparisons included 16 participants, but all other measures did not include that participant because of missing data at baseline (n=15).

^c^Italicized values indicate a significant *P* value <.05.

^d^EMIS: Expression of Moral Injury Scale.

^e^WHOQOL: World Health Organization Quality of Life.

### Qualitative Feedback

In the qualitative interview, participants provided feedback regarding the helpful features of MOVED as well as potential revisions to increase the perceived effectiveness.

#### Positive Feedback

First, positive feedback included noticeable positive changes in veterans across a wide range of domains, including behaviors, cognitions, emotions, and social functioning. Consistent with behavioral tendencies in the elevation literature, veterans reported changes in wanting to help and connect with others, including desires to volunteer and imitate virtues demonstrated in the videos (eg, helping homeless veterans). Noted cognitive changes included an increased awareness of the positive aspects of self and others, increased self-reflection, and positive shifts in perspective (eg, shifting from negative to positive and cognitive reappraisal of negative events). Sample responses within this theme are as follows:

The exercises in the program just made me more aware of how I [was] going through life not noticing the little goods in people or in myself. And I guess ultimately, that’s a good thing that I’m forced to think about it.

Woman aged 33 years

There is a problem when you constantly relive your military experience, you relive them through a certain perspective. And when you are in the study, it kind of gives you the ability to analyze that perspective and look over it. Kind of look at it in a new light.

Man aged 48 years

I had a hatred for people for a very, very long time. I think this is what got me back to sort of seeing the good in folks that’s out there, you know? I say this taught me to be loving towards other people again.

Man aged 37 years

Regarding emotional changes, veterans reported improved emotion regulation and decreases in negative emotions (eg, anxiety, irritability, anger, and disappointment):

I encountered something not too long ago that normally is out of character for me, but I caused a little situation, an individual got mad at me, and I was like, ‘My bad. I’m sorry.’ And their attitude completely changed. And normally that would have been maybe me breaking the guy’s skull open or something, you know in my old days. But it was like, you know what, I was in the wrong. Sorry.

Man aged 37 years

I’m a little more mellow and a little more—I wouldn’t say relaxed—but I’m able to not be bothered by things.

Man aged 48 years

I’m not as angry or point fingers at myself you know?...I wasn’t disappointed at myself for the things I’ve seen or have had to do.

Woman aged 50 years

Veterans also described increases in positive emotional experiences (eg, “Like the Grinch, my heart, it was a little warm. I could feel. It made me feel something positive, it wasn’t negative.”), including feeling more empathy, gratitude, and humility. Importantly, changes in social functioning were also noted, including being more engaged with family, improved relationships and communication with both family and coworkers, and greater social awareness regarding how one might be perceived by others and their capacity to have an impact on others:

Even with my children, they wouldn’t be as interactive, but now it’s different.

Man aged 41 years

I can have interactions with people and not just hate them automatically...Instead of just pushing on by, I should try to engage with people and not just be a loner for the rest of my life.

Woman aged 33 years

I actually talk to friends here and I’ve reached out to people that I haven’t talked to in years.

Woman aged 50 years

It helped me engage a little bit more with my daughter.

Woman aged 43 years

It helped, I guess, build a better bond with my son...and start sharing a little bit of things with him.

Man aged 37 years

I’ve noticed that my relationships with co-workers have improved tremendously.

Man aged 36 years

I used to do things without [my wife] and now, after seeing the first video, I told her I wanted to start doing workouts with her, so we started at least walking and jogging. This study has pushed me to do more things with her.

Man aged 43 years

#### Negative Feedback

A few veterans described negative experiences with MOVED, such as difficulties with the recall exercises and feeling less engaged in the second half of the intervention. In addition, some reported negative reactions to exercises such as exacerbating feelings of disconnectedness or ruminating on negative thoughts. Within this select subgroup, it should be noted that there was 1 outlier participant who reported no changes and even some negative reactions to the overall intervention. This veteran (aged 36 years) attributed his negative reaction to a contentious relationship with his identified significant other along with major cultural differences. He noted that because of his culture or race (self-identified as Black) and life experiences, he found it difficult to relate to the elevation induction exercises, which led to reluctance to complete the sessions because he did not think they would be helpful:

As a Black male, like I don’t really see a lot of that kind of stuff [referencing video content] in my community you know? Like that kind of stuff doesn’t happen. And so, for me, it looked like White people doing their stuff that don’t happen to us.

I mean to go further than the videos just themselves, the cultural differences, like those barriers, made it difficult for me to be able to see that happening in my day-to-day life. For it to be relatable.

Man aged 36 years

#### Suggested Revisions

##### Overview

All veterans also suggested some revisions to improve MOVED. One of the emergent themes was to integrate an explicit focus on negative experiences, such as traumatic and morally injurious events, to assist with processing negative experiences while also fostering positive experiences. Regarding the overall format, veterans suggested incorporating a military perspective throughout by using more military terms (eg, when defining virtues), structuring sessions and exercises like “missions,” and creating clear objectives for each week to help orient veterans to upcoming tasks:

I think the best way to go about it is just go by the military definitions of things because that’s what most people already understand. You know, you look at the army core values or the marine core values, things of that nature.

Man aged 37 years

I think if we established more of a battle rhythm, it would have been helpful. When I went on a mission, they told me about the objective, how we get there, okay let’s come back safe. You know, where this could be like, hey look for kind acts, look for good people, think about it, remember it, so you can report about it.

Man aged 43 years

Regarding treatment length, veterans advocated for additional flexibility by suggesting that there should be options to continue the treatment beyond 8 sessions or booster sessions should be made available. Similarly, it was suggested that creating the flexibility to choose 1 or 2 sessions per week (rather than making everyone complete 2 sessions) might benefit veterans with differing work schedules and personal responsibilities.

##### Video Revisions

In terms of specific session content, veterans remarked that changes to the videos, recall exercises, and goals could further enhance the treatment. First, veterans who noted negative reactions to the videos recommended avoiding “cheesy” videos and encouraged using more videos that veterans can connect to or would find more relatable. Another common revision suggested was to change the way in which the videos were distributed across the program by either adding more videos as a component of the standard intervention, alternating between video and recall exercises (ie, changing the distribution schedule), or adding extra or supplemental videos for use when needed. This recommendation was largely driven by veterans who described enjoying the videos but felt that they were presented too infrequently.

##### Recall Exercise Revisions

Additional recommendations for the recall exercises focused on expanding the target of noticing virtuous behaviors to include more options. For example, the prompt encouraged veterans to recall something they witnessed in the past few days that may have elicited elevation in themselves; however, several veterans noted challenges with completing this task and suggested that the treatment encourage veterans to search existing media to find acts of virtue or to recall any previous event in their life that may have similarly elicited an elevation-like response. Consistent with other feedback regarding the helpfulness of reminders, several veterans also encouraged sending text or email reminders to participants during the days when they are asked to look for virtuous behaviors.

##### Goal-Setting Revisions

An early suggestion for the goal-setting portion of MOVED, which was later endorsed by nearly all veterans, was to create a list of predetermined goals that might align with each induction exercise and allow veterans to choose the goal they would like to pursue from that list. Veterans suggested that this might reduce the burden of creating a brand-new goal and would likely increase engagement with the exercise. In addition to difficulties in creating a new goal, veterans reported challenges with remembering what goal was set and, in some cases, forgetting to complete the goal. Accordingly, they recommended adding text or email reminders that include the specific goal selected and task due the next session. Some veterans also suggested limiting goal setting to once per week (vs twice per week, 1 goal for each session) to increase the feasibility of completing that goal.

##### Therapist Involvement

A common theme among most veterans was that integrating some interactions with a therapist or another person would be helpful in conjunction with the current content, and some explicitly described wanting more human interactions. Suggestions for the potential involvement of a therapist ranged from optional, occasional consultation to scheduled weekly conversations with a therapist. Veterans indicated that they would have consulted a therapist to seek their help with identifying additional coping tools, processing emotions, processing traumatic or morally injurious events, and navigating goal-directed activities (eg, enhancing social connection).

##### Accessibility to Materials

Finally, the desire to access MOVED materials more easily during and after the intervention emerged as a theme. For example, veterans recommended providing video links in addition to displaying the videos within sessions, which would allow participants to watch the videos again or share them with others. Similarly, participants stated an interest in receiving access to previous session’s content for review, including psychoeducational materials, as well as information about what their previous goals were and their progress with the goals. Finally, some veterans requested the provision of feedback about participants’ progress during the course of treatment, which could include information regarding goals and recall exercises and results from self-report questionnaires about emotions and social interactions experienced during treatment.

## Discussion

### Principal Findings

A few extant studies imply the possible benefits of experiencing elevation for veterans with PTSD symptoms and moral injury distress. However, no study has tested an intervention using elevation to target these concerns in this population. The purpose of this mixed methods study was to conduct a pilot trial of a web-based elevation intervention to assess the overall feasibility, acceptability, and potential changes in targeted outcomes. Overall, the findings suggest that the MOVED intervention was largely feasible and that veterans found it acceptable and satisfactory as a treatment. In addition, veterans who completed MOVED reported large decreases in PTSD symptoms and a moderate increase in quality of life.

### Feasibility, Acceptability, and Satisfaction

First, recruitment and retention data suggest that participation in this study, the MOVED intervention condition in particular, was largely feasible. In addition, we found evidence for moderate-to-high levels of participation and retention for the MOVED sessions. For example, 79% (19/24) of veterans completed ≥4 sessions, with the majority completing at least 7 sessions (16/24, 67%). However, a limitation of this study is the retention of veterans at the follow-up survey for the treatment condition: one-third of veterans randomized to the treatment condition were missing posttreatment data, which could have led to biased results and should be addressed in future replication studies.

Overall, the quantitative and qualitative results suggest that veterans found MOVED to be acceptable and satisfactory at the overall treatment level, and participants reported high scores for helpfulness and engagement at the session level. Veterans who completed the treatment also highlighted several ways in which it could be improved, including integrating the processing of negative experiences, incorporating more military structure and terminology, increasing the flexibility of the treatment structure and duration, and ensuring that materials are culturally appropriate to veterans who identify as racial or ethnic minority individuals. Participants also identified specific improvements to the videos, recall exercises, and goal setting. Finally, participants suggested providing increased access to treatment materials throughout the intervention, additional accountability and feedback on progress, and some degree of integrated therapist interaction.

### Elevation Responses

Surprisingly, veterans in the MOVED condition endorsed lower state elevation scores following inductions than expected. A potential reason could be deficits in the measurement tool for state elevation, particularly with regard to the associated physical sensations. At the time of this study, there was no standard or widely used scale for state-level elevation with strong psychometric properties. However, a novel measure was recently developed and validated in both clinical and nonclinical samples [53]; therefore, future trials and other elevation-based studies should aim to use this validated measure to clarify these findings. Alternatively, there may be a ceiling effect for this sample population such that the individuals in this population do not experience this emotion or its physiological markers as strongly as those in other populations that are typically studied in elevation research (eg, nonclinical samples and civilian samples). It remains possible that PTSD or even military culture norms constrain the experience and expression of some positive social emotions.

Notably, there is some discrepancy between the elevation scores and other responses in the treatment condition. For example, veterans reported low-to-moderate elevation scores after the induction exercises but high scores for engagement and perceived helpfulness of the exercises for the same sessions. This difference could further highlight the limitations of the measure used. It could also suggest that veterans perceived benefits to the sessions regardless of whether they experienced a strong elevation response. The role of elevation responses following the induction exercises should be explored in future research to clarify the potential mechanisms of action if participation in this treatment truly leads to meaningful improvements in the targeted outcomes.

Moreover, the qualitative interviews suggested variability in individual responses to elevation inductions. This was highlighted by the contrast between veterans who described the videos as highly inspiring and even asked for more such videos compared with those who described the same videos as “cheesy” or unrelatable. The possibility of diverse reactions to video stimuli was underscored by a veteran sharing how his personal experiences and cultural background made it challenging to relate to the people in the videos, thereby preventing a strong elevation response. An important direction for this area of research is to better understand who is likely to respond to *what* elevation stimuli. Future work should aim to develop idiographic approaches with a more diverse set of elevation-eliciting stimuli, which could help tailor videos to the specific preferences and background of a given veteran. Such personalization may be important in general for efforts toward using elevation as a therapeutic tool.

### Targeted Outcomes

Veterans who completed MOVED reported a large decrease in PTSD symptoms. Although the small sample size for this pilot trial is not powered to test true treatment effects through group comparisons, the preliminary results of the MOVED condition are noticeably distinct from those of the control condition. Specifically, the MOVED group endorsed a large within-person decrease in symptoms (Cohen *d*=1.44), nearly twice that endorsed by the control condition (Cohen *d*=0.78). This finding is remarkable, given that the intervention did not directly or explicitly target trauma memories or distress symptoms. Nonetheless, the large effect within the MOVED condition paralleled veterans’ qualitative reports of positive pre-post changes in behaviors, cognition, and emotions consistent with trauma recovery (eg, increased social engagement, perspective shifting, and emotion regulation). In addition, the treatment condition reported medium-sized increases in physical and psychological quality of life, in contrast to the control condition, which reported no meaningful changes.

Unexpectedly, veterans in the MOVED condition did not endorse any change in their moral injury distress, whereas those in the control condition endorsed a medium-sized decrease in the total score (not subscale scores). It is unclear why the control condition would report decreased distress, other than random error, regression to the mean, or perhaps assessment reactivity—a reduction in distress because of completing self-report measures and reflecting on symptoms without the influence of the intervention [54]. However, qualitative feedback suggests that veterans in MOVED could have benefited from explicit engagement with moral injury distress during the treatment, making the link between session activities and features of moral injury clearer. Future trials should attempt to incorporate moral injury topics into the session content to facilitate direct integration alongside elevation content.

Finally, veterans in neither condition displayed changes in prosocial behavior. It is unclear why this might be the case, but if replicated with a larger sample, a null finding might suggest that (1) perhaps the elevation dose was too mild to elicit prosocial motivations, (2) perhaps participants’ session goals did not sufficiently target helping others (ie, more focus on self-improvement), or (3) perhaps contextual factors such as social distancing during the COVID-19 pandemic limited opportunities for prosocial acts. In addition, when considering findings for PTSD symptoms and quality of life, perhaps acting on motives for prosocial behavior may not be essential to attain benefits from MOVED.

### Limitations

Given the pilot trial’s aim of testing feasibility and acceptability, statistical tests in this small sample should be interpreted with caution, particularly for the targeted outcomes. Future studies with larger samples that also account for external factors (eg, engagement with other psychosocial or psychiatric treatments) are needed to replicate pre-post effects and formally test differences between groups that could be attributed to MOVED. Second, the state elevation measure did not perform optimally in this sample; accordingly, future studies should use the newly validated measure to further ensure the appropriate assessment of elevation at the state level. Third, despite substantial efforts, we were unable to obtain feedback from participants who dropped out or failed to complete the required sessions, and we could not inquire about their experiences with the intervention. Therefore, the initial pilot data are limited to those who completed the intervention. Future research should make additional efforts to explore the specific barriers and struggles for the subset of veterans who were unable to fully participate.

### Conclusions

Despite these limitations, this study has several strengths with potential clinical implications. First, this web-based intervention was easy to access. In addition to descriptive statistics showing engagement, qualitative responses highlighted the ease and benefits of accessing this treatment alone, with one’s own devices and with scheduling flexibility. Thus, this approach might be useful for targeting some of the known barriers to initiating and staying engaged with trauma-focused treatment [14-16]. This novel, accessible intervention also appeared to impact both PTSD symptoms *and* quality of life—important objectives, given the struggles of many veterans and findings that suggest patients rate functional aspects of recovery and improved quality of life as top targets for seeking mental health care [55,56]. Following replicated findings, MOVED might serve as a stand-alone package or a supplement to traditional trauma-focused treatments, emphasizing a unique focus on strengths and inspiring moral exemplars.

Overall, the findings provide preliminary evidence that veterans with PTSD and moral injury distress were interested in an intervention based on exposure and engagement with experiences of moral elevation. Future studies must further test and refine this novel approach for targeting trauma-related distress and quality of life, with the ultimate aim of increasing treatment access and inspiring veterans to improve their mental, social, and physical well-being.

## Abbreviations

CONSORT: Consolidated Standards of Reporting Trials
CSQ-8: Client Satisfaction Questionnaire-8
MINI: Mini-International Neuropsychiatric Interview
MOVED: Moral Elevation Online Intervention for Veterans Experiencing Distress Related to Posttraumatic Stress Disorder and Moral Injury
OSF: Open Science Framework
PCL-5: Posttraumatic Checklist for Diagnostic and Statistical Manual of Mental Disorders, Fifth Edition
PTSD: posttraumatic stress disorder
TEI-SF: Treatment Evaluation Inventory-Short Form

## Data Availability

The data set generated during this study is not publicly available because of legal restrictions and institutional policies surrounding data that contain potentially identifying and sensitive patient information. The minimal data set underlying the results described in the manuscript may be made available upon request from the corresponding author pending appropriate institutional approval.
